# Football behind bars: psychosocial mechanisms of wellbeing and social integration in a prison-based sport programme

**DOI:** 10.1108/IJOPH-03-2026-0037

**Published:** 2026-08-07

**Authors:** Jose Coto-Lousas, Carlos Suárez, Pablo Uría-Valle, Jacob Sierra-Diaz, Javier Fernandez-Rio

**Affiliations:** Department of Health Sciences, Public University of Navarre – Arrosadia Campus, Pamplona, Spain; Department of Education, University of Oviedo, Oviedo, Spain

**Keywords:** Prison, Social integration, Qualitative research, Football, Psychosocial wellbeing, Sport-based programmes

## Abstract

**Purpose:**

This study aims to examine the subjective experiences of people living in a Spanish medium-security correctional facility who participated in a structured football programme over an academic year, exploring how participation contributed to wellbeing, personal development and the creation of meaningful psychosocial spaces within prison.

**Design/methodology/approach:**

A qualitative design was used with 25 people living in prison (22 men and 3 women). Data were collected through semi-structured individual interviews, focus groups and non-participant observation, and analysed using reflexive thematic analysis.

**Findings:**

Three interconnected themes were identified: wellbeing, personal development and environment. Football was experienced not merely as a recreational activity but as a distinctive psychosocial space that promoted emotional regulation, social connectedness, positive identity reconstruction and weekly rituals that extended the programme’s influence beyond the sessions themselves, helping explain how football-based programmes may support rehabilitation in custodial settings.

**Research limitations/implications:**

This study was conducted in a single medium-security facility in Spain and included only three women, limiting transferability and gender-specific interpretation. Future research should include larger female samples, longitudinal designs and comparisons across correctional contexts.

**Practical implications:**

Structured football programmes may strengthen prison rehabilitation when delivered regularly, facilitated by staff with psychosocial as well as technical competencies, and designed to maximise meaningful participation and equitable access.

**Originality/value:**

To the best of the authors’ knowledge, this is the first qualitative study of a football-based intervention in a Spanish correctional setting. It extends the international literature by demonstrating football’s temporal, social and symbolic role as a psychosocial resource, using a multimethod design integrating interviews, focus groups and observation.

## Introduction

Prisons are highly regulated institutional environments in which surveillance and control substantially restrict individuals’ autonomy and capacity for self-determination, with significant implications for identity formation and emotional wellbeing (Liebling and Arnold, 2004; Moran *et al.*, 2023). The architectural design, organisational structures and normative frameworks governing daily life shape patterns of social interaction and influence perceptions of dignity, fairness and interpersonal respect (Reeves, 2016; Zubiaur-González, 2017). Emerging evidence indicates that both structural conditions and social dynamics within correctional institutions significantly shape opportunities for wellbeing and human development (Herold *et al.*, 2023; Koddebusch *et al.*, 2025). In response, several penal systems have progressively moved away from compliance-oriented and punitive models towards approaches grounded in rehabilitation, personal growth and social resilience (Kraft *et al.*, 2025; Mutz and Müller, 2023), positioning mental health promotion as a strategic priority (World Health Organization, 2024).

Positive mental health extends beyond the absence of psychopathology and encompasses psychological and social flourishing, including positive affect, a sense of meaning and constructive interpersonal relationships (Diener *et al.*, 2018; Westerhof and Keyes, 2010). Nonetheless, people living in prison consistently report elevated levels of anxiety, depressive symptomatology and physical decline compared with the general population, particularly where access to purposeful activity is restricted (De Vita *et al.*, 2019; Hilpisch *et al.*, 2023). Within this context, structured physical activity and sport initiatives have demonstrated potential not only to enhance physical and emotional health, but also to strengthen social connectedness, self-worth and motivation for behavioural change (De Marco and Meek, 2022; Dransmann *et al.*, 2024; Liguori and Calella, 2024; Meek and Lewis, 2014).

Self-determination theory (Ryan and Deci, 2000) offers a robust explanatory framework for understanding the psychological benefits of sport participation. When social environments support the basic psychological needs for autonomy, competence and relatedness, individuals are more likely to experience self-determined forms of motivation associated with sustained engagement and enhanced wellbeing. Within correctional settings, well-structured sport programmes may provide opportunities for mastery, emotional regulation and collective belonging that satisfy these needs (Dransmann *et al.*, 2024; Turner *et al.*, 2022). In parallel, social capital theory (Coleman, 1988; Putnam, 2000; Zubiaur-González, 2017) suggests that such initiatives may reinforce cooperative norms, mutual trust and shared responsibility, thereby contributing to a more constructive social climate within the institution. Likewise, social identity theory (Tajfel and Turner, 1979) proposes that shared group membership can strengthen feelings of belonging and positive self-concept, providing an additional framework for understanding the collective and relational dimensions of football participation in prison.

However, although self-determination theory clarifies motivational dynamics, it does not fully explain how participation in sport translates into sustained psychosocial transformation in prison contexts. To address this gap, the present study is informed by the Sport-Based Prisoner Rehabilitation Model (Morgan *et al.*, 2020), a framework developed specifically for correctional environments. This model posits that structured sport engagement can generate transformative experiences such as personal accomplishment, enduring commitment and the development of meaningful interpersonal connections which operate as mechanisms of change. These experiences may cultivate relatively stable personal assets (psychological capital), including self-efficacy, resilience and self-regulation, alongside transferable competencies (human capital) such as teamwork, discipline and accountability. Together, these resources are associated with improved psychological adjustment, constructive institutional behaviour and enhanced readiness for social reintegration.

The combined use of self-determination theory (SDT) and the sport-based prisoner rehabilitation (SBPR) Model responds to a specific analytical gap that neither framework addresses in isolation. SDT specifies when sport participation becomes motivationally meaningful, when basic psychological needs are satisfied, but does not explain how those motivational states translate into rehabilitation-relevant outcomes. The SBPR Model addresses precisely this translational question, mapping the pathway from sport-derived experiences to psychological and human capital. Together, the two frameworks operate at complementary levels of analysis that no single existing framework integrates simultaneously.

In line with this perspective, sport participation may facilitate the internalisation of prosocial values and reinforce commitment to rehabilitation and community re-entry (Youssef, 2024), while encouraging healthier and socially responsible lifestyles both during incarceration and following release (Dransmann *et al.*, 2024; Koddebusch *et al.*, 2025; Kraft *et al.*, 2025). From a socio-ecological standpoint, programme structure, relationships with coaches and peers and broader environmental conditions interact dynamically to shape participants’ experiences, thereby amplifying the potential impact of sport on psychological health and overall quality of life (Luthar *et al.*, 2000).

The Spanish correctional context provides a particularly relevant setting in which to examine football-based interventions. Spanish correctional policy formally emphasises rehabilitation and social reintegration as the primary objectives of imprisonment, as established in the Spanish General Penitentiary Act (Ley Orgánica 1, 1979; hereafter LOGP) and further developed in the Prison Regulations (Real Decreto 190, 1996), with sport recognised as one component of educational and social intervention programmes within correctional institutions (Martí & Cid, 2015). At the same time, football occupies a particularly prominent cultural position in Spanish society, where participation and spectatorship extend across age groups and socio-economic backgrounds (Llopis-Goig, 2015). This widespread cultural familiarity may enhance participants’ identification with football-based programmes, increasing their perceived relevance and their potential to facilitate social connection, personal meaning and engagement during incarceration.

Within this Spanish context, football represents a particularly promising intervention. Beyond being Spain’s most popular sport, football is deeply embedded within everyday social life and collective identity, making it especially well suited to generate meaningful engagement among people living in prison. Compared with individual exercise modalities frequently implemented in prisons, such as fitness training, running or gym-based programmes (Herold *et al.*, 2023; Meek and Lewis, 2014), football requires continuous cooperation, shared tactical decision-making and collective responsibility. These characteristics may intensify relational dynamics and create stronger opportunities for social bonding and identity negotiation within custodial environments (Dransmann *et al.*, 2023; Morgan *et al.*, 2020). Consequently, football-based interventions may activate distinct psychosocial processes compared with more individually oriented forms of physical activity (Putnam, 2000; Zubiaur-González, 2017). Its practice promotes intensive interaction and shared endeavour, which can strengthen interpersonal ties and foster a sense of collective identity (Dransmann *et al.*, 2023; Ortega Vila *et al.*, 2020). Engagement in training sessions and competitive matches offers opportunities for both individual and collective achievement, facilitates the development of prosocial competencies such as empathy and communication, and provides a constructive outlet for frustration and aggression, thereby contributing to reduced conflict and a more positive institutional climate (Herold *et al.*, 2023).

Previous research supports these assumptions. Martín-González *et al.* (2018) reported that a football-based intervention enhanced inter-group relations among participants, increasing empathy and reducing prejudice. More broadly, qualitative research by Jacobs and Wahl-Alexander (2021), Meek (2014) and Norman *et al.* (2024) has highlighted the capacity of sport-based programmes to create opportunities for personal growth, social connection and psychosocial development within correctional settings. More recently, Newson *et al.* (2024a) found that a soccer programme fostered social connectedness, improved behavioural conduct within prison and supported reintegration processes. Two complementary qualitative studies from the same programme extend these findings in directions directly relevant to the present work. Newson *et al.* (2024b) examined the experiences of people serving community sentences enrolled in the Twinning Project, finding that football’s capacity to bridge institutional and community identities was central to its rehabilitative potential. Peitz *et al.* (2025) focused specifically on women in the same programme, documenting positive outcomes (including improved self-perceptions and prosocial intentions) alongside structural barriers to engagement such as programme design constraints and limited perceived control over post-release outcomes. The present study extends this body of work by examining a Spanish custodial context outside the Twinning Project model, combining three data collection methods rather than interviews alone, and including a mixed-gender sample within a single programme.

Nevertheless, despite this growing body of evidence, research specifically examining football interventions in prisons remains limited. Existing qualitative evidence is concentrated largely within Anglophone correctional systems, whereas little is known about how football is experienced within Southern European prisons, where institutional organisation, rehabilitation policies and the cultural significance of football differ substantially. Moreover, previous qualitative studies have relied primarily on interview data. The present study addresses these gaps by combining semi-structured interviews, focus groups and systematic observation to generate a richer understanding of participants’ lived experiences within the Spanish correctional context.

Furthermore, much of the existing research involving people living in prison has relied predominantly on quantitative methodologies and self-report questionnaires (De Vita *et al.*, 2019). Given the particular characteristics of some people living in prison, such as limited literacy or psychological vulnerability, several scholars have advocated for qualitative approaches (Llorach-Segalà *et al.*, 2025). Such methods provide richer, context-sensitive accounts of the mechanisms through which sport may influence identity construction, emotional regulation and interpersonal relationships in prison.

Against this background, the present study examines the psychosocial mechanisms activated through sustained participation in a structured football programme within a Spanish correctional institution. Drawing on the Sport-Based Prisoner Rehabilitation Model, the research explores how engagement in a collective sport environment may contribute to processes of emotional regulation, identity reconstruction and social integration among people living in prison. By extending the limited qualitative literature on football in prison to the Spanish correctional system and by adopting a multimethod qualitative design, the study seeks to provide a deeper understanding of how football-based interventions may function as settings that promote psychological wellbeing, relational development and rehabilitation within custodial environments.

## Design and participants

The present study used a qualitative research design aimed at developing an in-depth and context-sensitive understanding of the experiences of people living in prison participating in a football-based sport programme. This methodological approach is particularly appropriate when the objective is to explore lived experience, perceived processes and attributed meaning, enabling analysis of the dynamic interplay between individual experiences, group interactions and the broader institutional environment (Colorafi and Evans, 2016). The choice of design is consistent with prior research on sport interventions in prison contexts, which has emphasised the value of qualitative inquiry in identifying mechanisms of change that extend beyond quantitative outcome indicators (Gallant *et al.*, 2015; Llorach-Segalà *et al.*, 2025).

The sample comprised 25 individuals (22 men and 3 women) residing in a single medium-security Spanish correctional facility located in Northern Spain. The majority were serving medium- to long-term sentences (ranging from 3 to 20 years), and the group included individuals convicted of both violent and non-violent offences. All participants were regular attendees of the football programme and had engaged in the intervention for a minimum of six consecutive months, ensuring sustained exposure rather than sporadic participation. The programme consisted of weekly training sessions and organised matches conducted throughout a one-year period within the institutional sports facilities.

Participants were selected through criterion-based purposive sampling. To be eligible for inclusion in the study, participants were required to:

- be actively enrolled in the football programme;
- have participated regularly for a minimum of six consecutive months; and
- have accumulated sufficient experience within the programme to provide meaningful reflections on their participation.

Participation in the football programme itself was open to all people living in prison, regardless of age, sentence length or type of offence. However, eligibility and access to the programme were managed by the prison administration according to its internal procedures. This sampling strategy sought to capture perspectives grounded in prolonged engagement with the intervention rather than initial impressions.

The three female participants were retained in the sample on both methodological and substantive grounds. In reflexive thematic analysis, the value of qualitative accounts is determined by contextual depth and specificity, not by numerical thresholds (Braun and Clarke, 2006). A dedicated women-only focus group was conducted to ensure female participants’ perspectives were not subsumed within the dominant male accounts. Their data revealed a qualitatively distinct experiential pattern, centred on access, recognition and institutional exclusion, that constitutes a substantive contribution to the study’s findings. The limitations of this small female subsample for gender-comparative analysis are addressed explicitly in the Limitations section.

To safeguard anonymity within a relatively small and identifiable institutional population, detailed individual-level sociodemographic and criminological profiles were not systematically recorded, in line with ethical recommendations for research involving vulnerable groups (Orb *et al.*, 2001). Consequently, individual information such as participants’ exact ages was not collected or reported, as this could increase the risk of identification within the correctional setting. However, broad contextual indicators (e.g. age range, general sentence duration category and offence type classification) were retained to enhance the transferability of findings without compromising participant confidentiality.

This level of contextual description enables readers to assess the potential transferability of the findings to comparable custodial settings, consistent with qualitative criteria of transferability as outlined by Guba and Lincoln (1989).

## Procedure

Before the commencement of the study, the prison management team and the staff responsible for the sport programme were informed of the study aims, methodological framework and participation requirements. Institutional authorisation was granted by the Spanish Ministry of the Interior, the authority responsible for the correctional institution, and ethical approval was obtained from the University Research Ethics Committee (reference: 20_RRI_2023). The study was conducted in accordance with the principles of the Declaration of Helsinki (World Medical Association, 2013) and with national and international guidelines governing research involving vulnerable populations. The research focused on a year-long football-based intervention implemented within the institutional sports programme.

Recruitment was undertaken on a voluntary basis, without direct involvement of prison staff in the final selection of participants, to minimise potential perceptions of coercion or institutional pressure. Researchers met prospective participants in person to provide detailed information regarding the nature and purpose of the study, emphasising their right to withdraw at any stage without adverse consequences for their institutional status or involvement in the sport programme.

Written informed consent was obtained only after participants had received both oral and written information about the study. Particular attention was paid to minimising potential power imbalances inherent in prison research. The participation was entirely voluntary. Confidentiality was ensured through systematic anonymisation and the removal of any potentially identifying information. All records were securely stored and accessible only to the research team. Data collection was conducted midway through the annual programme to capture reflections from participants who had already accumulated substantial experience within the intervention while still incorporating perspectives from more recent participants. In addition, participants were explicitly informed that their decision to participate or decline would have no influence on their institutional status, privileges or access to this programme. Of the 32 individuals invited through informational posters and briefing sessions in the sport unit, 25 agreed to participate (78% acceptance rate), with no subsequent withdrawals.

## Instruments

Data were collected using three complementary qualitative methods: semi-structured individual interviews, focus groups and non-participant observation. This multimethod strategy was adopted to facilitate data triangulation and to capture both individual experiences and collective dynamics (Patton, 2015).

### Individual interviews

Fifteen semi-structured interviews were conducted over a six-week period, each lasting approximately 40–60 min. Interviews were led by a member of the research team with formal training in qualitative methodology. This format enabled an in-depth exploration of participants’ interpretations of their involvement in the programme, including motivations for engagement, lived experiences and perceived effects across emotional, social, physical and personal development domains. Interviews took place in a designated room within the prison, with only the interviewer and participant present, to foster confidentiality and encourage open dialogue.

### Focus groups

Three focus groups were conducted between weeks four and six of the data collection phase (two mixed-gender groups and one women-only group). Each session lasted between 60 and 75 min and involved between seven and nine participants. The same researcher who conducted the interviews facilitated the discussions. This method enabled the identification of shared narratives, comparison of perspectives and intersubjective validation of emerging categories through group interaction (Krueger and Casey, 2015). The discussion guide was structured around the same thematic domains as the interviews, adapted to stimulate collective reflection and exchange.

### Non-participant observation

Non-participant observation was undertaken by a second member of the research team during weekly football sessions. The observer remained in the stands to minimise disruption and recorded structured field notes covering attendance, prosocial interactions, instances of conflict, expressions of enjoyment or relaxation and coach–participant dynamics. Following each session, an observation template was completed alongside a reflexive journal designed to identify and critically appraise potential researcher bias (Cohen *et al.*, 2011).

## Intervention programme

The programme under study was designed and implemented by an external sports foundation specialising in social inclusion through football. One member of the research team was responsible for delivering the sessions within the correctional institution as part of this institutional programme. The remaining members of the research team acted as independent evaluators with no involvement in programme design. The intervention spanned a full academic year (October to June) and comprised one 90-min session per week (36 sessions in total).

Each session followed a consistent format combining initial warm-up and group cohesion activities, ball-based technical drills aimed at the progressive development of skills, cooperative tasks designed to enhance communication and shared decision-making and adapted match play tailored to the abilities and characteristics of the group. This structure was designed to facilitate experiences of achievement, belonging and shared responsibility, elements identified as central to psychosocial change processes in prison sport programmes, particularly in relation to competence development, social bonding and the reinforcement of prosocial norms (Meek and Lewis, 2014; Morgan *et al.*, 2020).

Session planning accounted for spatial constraints, variability in physical competence and the need to encourage active participation, collective cohesion and emotional self-regulation. The coach-to-participant ratio was approximately 1:12, with an average attendance of 18 individuals per session. Adjustments were implemented for participants with physical limitations, and all activities adhered to institutional safety protocols.

Beyond its recreational dimension, the programme was conceptualised as a structured arena for meaningful social interaction, consistent with evidence identifying team sport as a vehicle for emotional wellbeing, social integration and the development of socio-emotional competencies within prison environments (Meek and Lewis, 2014; Morgan *et al.*, 2020).

## Data analysis

Data were analysed using reflexive thematic analysis with a predominantly inductive orientation, following the phases outlined by Braun and Clarke (2006). This approach enabled the identification of emergent patterns of meaning within participants’ accounts, while subsequently engaging in theoretical dialogue with the adopted conceptual framework. All interviews and focus groups were audio-recorded, transcribed *verbatim* and anonymised through alphanumeric coding. Files were stored on encrypted institutional servers in accordance with the approved ethical protocol.

The analytical process unfolded in several stages. First, repeated and immersive reading of the transcripts facilitated familiarisation with the data set. Second, an initial line-by-line coding process generated descriptive codes capturing meaningful units of data. These codes were progressively clustered into provisional themes through an iterative process of constant comparison and conceptual refinement. Once a preliminary thematic structure had been developed from the individual interviews, focus group transcripts and observational field notes were incorporated with two purposes: to elaborate and nuance the emerging themes and to examine their consistency across data sources. This strategy enriched the interpretation by enabling comparison across complementary sources of evidence and enhancing the contextual depth of the emerging themes.

In a subsequent interpretative phase, the final themes were examined through the lens of the Sport-Based Prisoner Rehabilitation Model, assessing the extent to which emergent categories could be understood as experiences fostering psychological capital and human capital, or as structural conditions enabling change. Two researchers independently engaged with the data set throughout the coding process before comparing and discussing their interpretations during a series of reflexive analytical meetings involving the wider research team. Two additional researchers subsequently reviewed the developing thematic framework and contributed to discussions concerning theme definitions, category boundaries and the interpretation of the data. These discussions were intended to deepen interpretative reflexivity, challenge emerging assumptions and enrich analytical understanding, rather than to establish inter-coder agreement. This collaborative process enhanced the credibility and dependability of the analysis by ensuring that the thematic structure reflected a critically developed and collectively refined interpretation of the data set (Lincoln and Guba, 1985; Sparkes and Smith, 2014). Throughout this analytical process, the researchers engaged in continuous reflexive dialogue to critically consider how their disciplinary backgrounds and previous experience in sport research might influence interpretation. A particular reflexive consideration concerns the dual role of one member of the research team, who served both as programme deliverer and as a co-analyst of the data. To mitigate the potential influence of this positionality on interpretation, all data collection (including individual interviews, focus groups and observational records) was conducted exclusively by other members of the team who had no direct involvement in programme delivery. Analytical decisions were made collectively across all four researchers, with the programme deliverer explicitly identifying and bracketing experiential assumptions during coding discussions (Sparkes and Smith, 2014). The analysis was supported by MAXQDA Analytics Pro 2022 (VERBI Software, Berlin, Germany).

To enhance analytical rigour, source triangulation was applied (Denzin, 1978), and an analytical matrix was developed linking emergent categories to their respective data sources (interviews, focus groups and observation) and to illustrative quotations. Trustworthiness was addressed in line with the criteria of credibility, transferability, dependability and confirmability proposed by Guba and Lincoln (1989), alongside specific recommendations for qualitative research in sport sciences (Sparkes and Smith, 2014). Each category was operationally defined to ensure interpretative consistency. For instance, accounts describing cognitive disengagement from the prison environment were grouped under the theme “Psychological escape”, whereas narratives referring to embodied enjoyment and post-activity relaxation were subsumed under “Post-activity satisfaction and relaxation”. The analytical process continued until sufficient interpretative depth had been achieved to address the research aims, with no substantively new patterns of meaning emerging during the later stages of analysis (Guest *et al.*, 2006). Representative quotations were selected to illustrate each theme while preserving the diversity and complexity of participants’ experiences across the data set.

## Results

The analysis generated three interconnected themes that illuminate how the football programme functioned as a psychosocial space within the prison environment: Wellbeing, Personal Development and Environment. Across interviews, focus groups and observational records, participants consistently described the programme as much more than a recreational activity, portraying it as a meaningful space for emotional regulation, social connection and personal growth. As shown in Table 1, most participants described improvements in emotional wellbeing, stronger interpersonal relationships and personal growth. For example, one participant explained the sessions allowed her to “disconnects me from problems” and “forget that I’m stuck in this s***” (FP-1), while observational records noted that “there’s constant affection between participants” (EO), highlighting the positive interpersonal climate that characterised the sessions.

**Table 1:** Categorisation of participants’ responses

Theme	Category	Label	Representative excerpts
Wellbeing	Emotional	Escape	“In the end, you’re not thinking about the argument you had yesterday or this morning, or about your sentence” (MP-5) “It clears my mind a lot; I feel like when I was a child running after the ball” (MP-8) “It disconnects me from problems, I forget that I’m stuck in this s***” (FP-1; FG-2)
		Self-esteem	“I feel better mentally. And my self-esteem also goes up” (MP-1) “I think I’m a little happier inside” (MP-9) “I feel good about myself” (MP-3; FG-3) “It helps me feel that I’m worthwhile, that I’m doing something right” (MP-10; FG-2)
		Satisfaction	“I like it because I learn things I didn’t know, I have fun like a child” (MP-7) “While playing football, honestly, I feel very comfortable and well” (MP-20) “We have a great time, and that brings us joy” (MP-4; FG-2) “Everyone, without exception, is happy, they have fun during the activities” (EO)
		Anxiety reduction	“It’s really good for my anxiety” (MP-5) “Honestly, it helps me a lot. It gives me peace, calm, it relaxes me” (MP-20) “We’re tense every day, and when we come here, we relax, play and support each other” (MP-22; FG-2)
	Physical	Relaxation	“It’s good to release endorphins” (MP-13; FG-1) “I feel very relaxed, like everything is gone” (FP-3) “I arrive tired at the cell, I feel grateful and comfortable with myself” (MP-8)
		Rest	“Afterwards I sleep well; I lie down in the barracks in the afternoon and sleep well” (MP-22; FG-2) “After playing, I’m more relaxed and sleep better” (MP-3)
	Social	Positive relationships	“You come here to have fun and be with your mates” (MP-1) “Happy to be with people you don’t see often, you know? And to talk” (MP-21) “There’s constant affection between participants” (EO) “You do what you like, and there’s a friendship and a good atmosphere” (MP-9; FG-3)
		Respect and coexistence	“Here there’s a lot of respect. Towards the coach and among ourselves” (MP-19) “Exercises are intense, but there’s a lot of respect between all participants” (EO) “In the module, there are fights and people get angry. Here, no, because it’s organised” (MP-11; FG-2) “You learn to be a better teammate, especially us (women)” (FP-3; FG-1)
Personal development	Growth	Overcoming	“I take it as a challenge, to improve something I’m not good at” (MP-9) “You try to improve yourself physically and mentally” (MP-11; FG-1) “Now I’m no longer embarrassed; I have confidence” (FP-3) “I improved a lot, I’m no longer ashamed and I’m not afraid because I know everyone now” (FP-1; FG-3)
		Learning	“I used to be really clumsy with my feet. And now… I think I’m doing okay” (MP-2) “During games, when there’s no ball, I don’t feel so clumsy” (FP-2) “Watching them, how they move and all, I watch and learn a bit more” (FP-1) “I like everything because with the exercises I notice I improve, and I like that” (MP-3)
		Commitment	“I’ve been in the programme for four years already” (MP-12) “Everyone makes an effort: high-skill and low-skill participants; all very motivated” (EO) “If I don’t do anything in a day, I feel like I’ve wasted it” (MP-7) “I like coming, you see, I never miss a session” (FP-2; FG-3)
	Integration	Humanisation	“Sometimes here we’re just a number, and they treat us like footballers, they teach us” (MP-8) “They care about each of us, whether we’re doing well or badly” (MP-8; FG-1) “It feels like a football class in any educational centre, like we’re students” (EO)
		Inclusion	“We suggested volleyball, but they didn’t allow it. They don’t accept anything for us (women)” (FP-1) “Without this, we would never play football” (FP-1; FG-1) “The treatment between men and women is excellent; women are fully integrated into activities” (EO) “They’re more used to us now; we’re more integrated” (FP-3; FG-1)
Environment	Conditions	Leaving the module	“Leaving the module a bit to clear my head” (MP-1) “I started to participate just to pass time, to get out of the module a bit” (FP-3) “Now coming here feels like leaving… leaving the module, having a laugh, being with people going through the same experience as me and distracting myself” (MP-9)
		Lack of alternatives	“Of course, if you don’t leave the module, the sentence feels twice as hard. I don’t do anything else” (MP-17) “Otherwise, we don’t go to any other activity” (MP-5) “It should be more days a week because it’s really good for prisoners” (MP-19)
	Rituals	Pre-session preparation	“The night before, you’re already thinking about the next day, preparing your clothes… everything” (MP-16; FG-2) “In the end, you prepare your clothes; it’s like leaving the routine here” (MP-5) “I like the fact that I have to prepare myself, I start laying everything out” (MP-21)
		Special day	“It’s the best day of the week” (FP-3; FG-2) “The day before, us girls in the module are all excited - tomorrow is Thursday!” (FP-1) “It’s the day you look forward to in the week” (MP-3; FG-3) “I’m already… already waiting for Thursday since Tuesday! Yes, yes, yes” (FP-3)

Note(s):

MP = Male participant; FP = female participant; FG = focus group; EO = external observer

**Figure 1:**
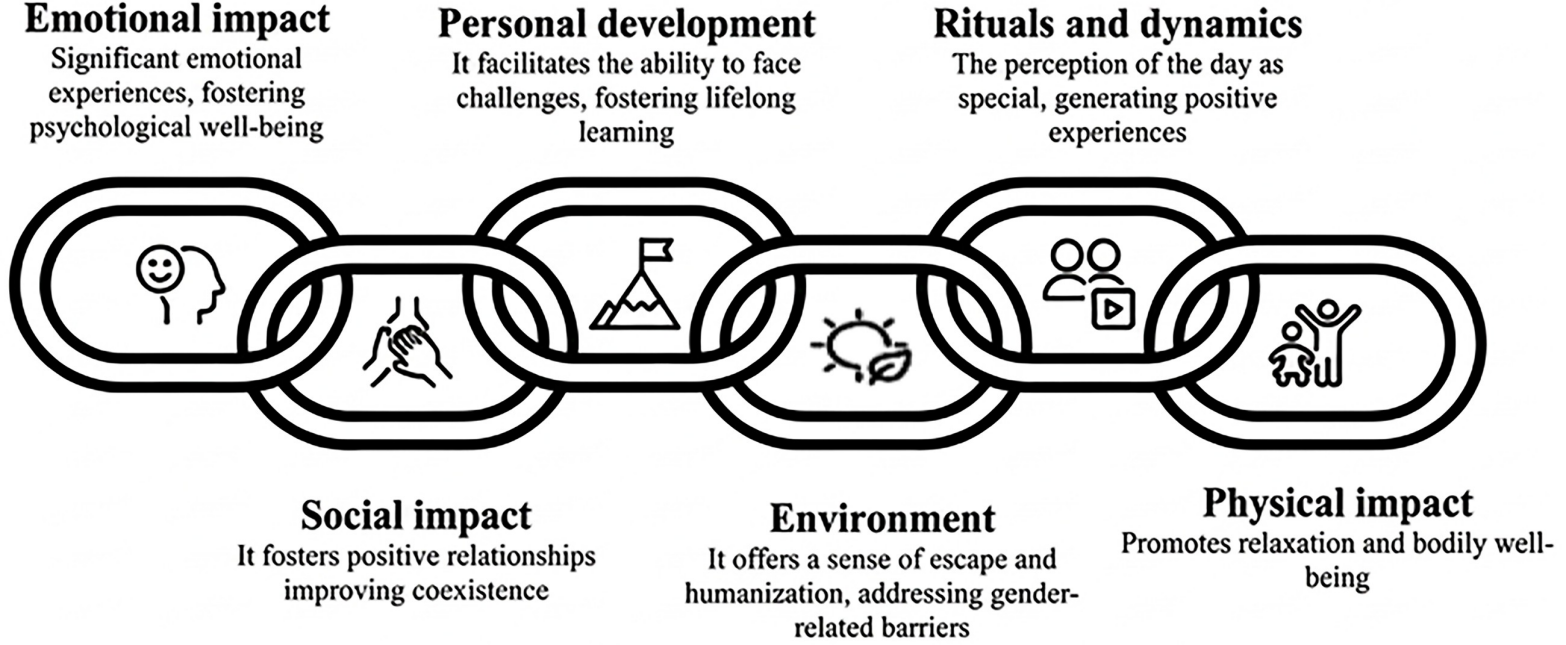
Thematic categories **Source(s):** Authors’ own work

Together, these findings suggest that football created opportunities not only for immediate enjoyment but also for psychological regulation, social inclusion and the development of more positive self-perceptions within the custodial environment. The thematic structure comprised 18 analytic labels derived from the triangulation of interviews, focus groups and observational data (Table 1), reflecting the dynamic relationship between individual experiences, processes of personal change and the institutional environment in which the programme was implemented (Figure 1). Across the three sources of data, the identified themes showed a high degree of convergence. No substantial contradictory perspectives emerged between interviews, focus groups and observational records; rather, the different sources complemented one another by providing additional contextual depth and reinforcing the credibility of the findings. Representative quotations are provided through the following section to illustrate the experience underpinning each theme.

### Wellbeing

This theme encompassed emotional, psychological and social dimensions of wellbeing. Collectively, participants described football as a mechanism of psychological regulation that allowed them to temporarily distance themselves from the prison routine, while fostering positive affective states and social connectedness (e.g. “It clears my mind a lot, I feel like when I was a child running after the ball” (MP-8); “We′re tense every day, and when we come here, we relax, play and support each other” [MP-22; FG-2]). Across interviews, focus groups and observational records, these experiences emerged consistently, suggesting that the programme was perceived not merely as an opportunity for physical exercise but as a meaningful psychosocial space within everyday prison life:

- *Emotional impact*. Most participants reported experiences of psychological escape, increased self-esteem, satisfaction and reduced anxiety. Several described football as an opportunity to disconnect from the prison environment and momentarily forget the constraints of incarceration (“I forget that I am stuck in here” [MP-5]). Participants also referred to enhanced self-worth and confidence through participation, with one stating that “my self-esteem also goes up” (MP-1). These accounts were accompanied by descriptions of enjoyment, comfort and psychological relief. Participants frequently portrayed football as one of the few moments during the week in which they could temporarily disengage from the emotional burden associated with imprisonment. Feelings of happiness, calmness and personal worth were repeatedly described, indicating that the activity created opportunities for emotional regulation beyond the immediate experience of playing football.
- *Physical impact*. Many participants highlighted feelings of relaxation and improvements in sleep quality following the sessions. Physical exertion was frequently associated with tension release and recovery from the stresses of prison life. As one participant remarked, “It’s good to release endorphins” (MP-13), whereas another reported that “afterwards I sleep well” (MP-22). Others described returning to their cells feeling physically tired but psychologically relieved, suggesting that the sessions facilitated both physical recovery and emotional decompression after prolonged periods of confinement. These narratives suggest that football contributed to post-activity physiological regulation and recovery. Collectively, participants experienced football not only as physical exercise but also as a means of relieving accumulated tension and restoring a sense of physical and psychological balance.
- *Social impact*. Participants consistently emphasised the social benefits of the programme. The sessions were perceived as spaces characterised by respect, cooperation and positive relationships, contrasting with the tensions often experienced elsewhere in the institution. One participant stated that “here there’s a lot of respect” (MP-19), while observational records noted that “there’s constant affection between participants” (EO). Several participants also described strengthened friendships and a greater sense of group cohesion. Interactions that would rarely occur within the ordinary prison routine became possible during the sessions, allowing participants to cooperate with others, communicate more openly and experience a sense of belonging within the group. Observational data reinforced these accounts by documenting frequent expressions of mutual support, encouragement and respectful behaviour throughout the activities. Together, these findings portray football as a structured social environment by cooperation and mutual respect, which participants contrasted with interpersonal tensions experienced elsewhere in the institution.

### Personal development

This theme comprised two interconnected processes: growth and integration. Together, participants described how sustained engagement in the programme promoted experiences of competence, confidence, social connectedness and the development of more positive self-perceptions within the prison environment:

- *Growth*. Some participants reported experiences related to overcoming challenges, learning and commitment. For example, one participant stated, “Now I’m no longer embarrassed; I have confidence” (FP-3), while another explained that “I take it as a challenge, to improve something I’m not good at” (MP-9). Participants also highlighted the satisfaction associated with skill development, with one participant noting, “I used to be really clumsy with my feet. And now […] I think I’m doing okay” (MP-2). Sustained commitment (long-term attendance and consistent motivation) was evident in accounts of long-term participation and consistent motivation, suggesting that engagement in the programme held personal meaning for many participants.
- *Integration*. Experiences of humanisation and inclusion were also relevant. Several participants highlighted the importance of being treated as “footballers” rather than as “numbers”, indicating a symbolic shift in social role. As one participant explained, “Sometimes here we’re just a number, and they treat us like footballers, they teach us” (MP-8). Although the present study was not designed to compare experiences according to gender, the accounts provided by the three female participants offered additional contextual insight into the particular significance of the programme for women within this correctional setting. Specifically, they described football as one of the few opportunities available for sport participation and social interaction. As one participant remarked, “Without this, we would never play football” (FP-1), while another noted that “they’re more used to us now; we’re more integrated” (FP-3). Given the small number of female participants, these observations should be interpreted as contextual insights rather than evidence of gender-specific differences. Nevertheless, they illustrate how access to football may hold particular relevance for women in correctional environments where opportunities for organised physical activity are limited.

### Environment

This theme encompassed contextual elements that shaped how participants experienced the football programme. Participants consistently described football as a distinctive break from the institutional routine, highlighting the importance of environmental conditions and weekly rituals in amplifying the psychological meaningfulness of sport participation:

- *Conditions*. Many participants described football sessions as an opportunity to leave the prison module and temporarily escape the monotony of daily confinement. As one participant explained, “Leaving the module a bit to clear my head” (MP-1), while another noted that participation initially served as a way of “passing time” and “getting out of the module a bit” (FP-3). Participants also emphasised the scarcity of alternative activities within the institution, which increased the perceived value of the programme. As one participant remarked, “If you don’t leave the module, the sentence feels twice as hard” (MP-17), highlighting the significance attributed to the weekly sessions.

Although participants evaluated the programme very positively, they also identified some limitations. Specifically, several participants expressed that one weekly session was insufficient and suggested increasing the frequency of the programme. As one participant expressed, “It should be more days a week because it’s really good for prisoners” (MP-19). Others indicated that the programme’s perceived value was partly explained by the limited availability of alternative recreational or educational activities within the prison. These accounts suggest that participants’ experiences should be interpreted not only in relation to the characteristics of the football programme itself but also within the broader institutional context in which opportunities for meaningful leisure and social interaction were scarce.

- *Rituals*. Some participants frequently referred to the anticipation surrounding football sessions, describing them as an important event within the weekly prison routine. One of them reported that preparing their clothes and equipment the evening before could transform the participation into a meaningful ritual (e.g. “The night before, you’re already thinking about the next day, preparing your clothes […] everything” [MP-16]). Several participants explained that this preparation began well before the session itself, generating positive anticipation and giving them something meaningful to look forward to during the week. These accounts suggest that the programme influenced participants’ daily lives beyond the time spent on the football pitch, becoming integrated into their weekly routines and expectations.

In the same vein, Thursday, the day on which the sessions took place, was repeatedly described as the most anticipated day of the week. Thus, one participant stated that “It’s the best day of the week” (FP-3), while another remarked that “It’s the day you look forward to in the week” (MP-3). For some participants, this anticipation emerged several days in advance, as reflected in the statement, “I’m already waiting for Thursday since Tuesday!” (FP-3). These narratives indicate that football sessions became temporal landmarks within prison life, providing a predictable and positive interruption to institutional routine. Rather than representing only a sporting activity, the programme appeared to organise participants’ perception of time and create shared moments of expectation and collective engagement.

Across these themes, participants consistently described the football programme as a distinctive social space within the prison environment. Rather than being experienced solely as a recreational activity, football functioned as a structured arena for psychological escape, emotional regulation and cooperative interaction. These processes suggest that sport participation may contribute to the development of psychological and relational resources that extend beyond the activity itself and potentially support broader processes of adjustment and rehabilitation within custodial contexts.

## Discussion

The aim of the present study was to examine the subjective experiences of people living in a Spanish medium-security correctional facility who participated in a structured football programme across a full academic year. The analysis identified three interconnected themes: wellbeing, personal development and environment, through which football participation operated as a meaningful psychosocial resource within the custodial setting. Taken together, the findings suggest that football’s contribution in this context operated not primarily through its technical content but through its structural role as a distinctive temporal, social and symbolic space within the prison environment. The Wellbeing theme reveals that the programme functioned as a mechanism of psychological regulation, providing people living in prison with a recurring opportunity to temporarily disengage from the emotional weight of incarceration, an experience that participants articulated not merely as distraction but as a qualitatively different mode of being within the institution. The Personal Development theme suggests that sustained participation created conditions for the emergence of more positive self-perceptions and social identities, through processes of mastery, recognition and collective belonging that the ordinary prison routine does not afford. The Environment theme represents one of the most distinctive findings of the study. Participants not only value the sessions themselves but the anticipatory rituals that structured their experience of the week around them, indicating that the programme’s psychosocial reach extended well beyond the 36 sessions into the fabric of daily institutional life.

These three themes are mutually reinforcing: the temporal structure created conditions for emotional regulation; emotional regulation facilitated personal development; and personal development was consolidated through the social recognition embedded in the football programme. Together, the findings are consistent with, and extend, the growing qualitative literature on sport-based programmes in correctional settings.

The first theme, *Wellbeing*, encompasses three interrelated dimensions: emotional, physical and social. Participants’ accounts of psychological escape (temporarily disengaging from the emotional burden of incarceration) are consistent with Norman and Andrews’ (2019) analysis of how sport participation in custodial settings enables people living in prison to construct alternative spatial and temporal realities, asserting limited agency over their experience of confinement. In the present study, this function extended beyond cognitive distraction: participants described a qualitatively different mode of being during sessions, one in which institutional identity and the weight of their sentence were temporarily suspended. This finding aligns with the observation by Gallant *et al.* (2015) that sport in prison simultaneously serves as a source of personal liberation and institutional management, a tension that is also evident in the conduct-contingent access structure of the present programme, discussed further below.

The social dimension of the Wellbeing theme is particularly salient. Participants consistently described the sessions as spaces of respect and cooperation that contrasted sharply with the interpersonal tensions prevalent elsewhere in the institution. These accounts are consistent with the findings of Dransmann *et al.* (2023) on the relational outcomes of football interventions in German prisons and with the evidence from Herold *et al.* (2023) indicating that team sport generates stronger prosocial outcomes than individual exercise modalities in custodial settings. From an SDT perspective (Ryan and Deci, 2000), these relational dynamics reflect the satisfaction of the need for relatedness, a need that the prison environment structurally suppresses and that football’s cooperative structure is particularly well suited to activate.

The physical dimension (post-activity relaxation, improved sleep quality and tension release) reflects the physiological regulation function of structured exercise documented across multiple prison sport studies (Kraft *et al.*, 2025; Mutz and Müller, 2023). These findings also speak to the specificity of football as an intervention modality. Compared with individually oriented exercise programmes frequently implemented in custodial settings (gym-based training, running or fitness circuits) football’s continuous cooperative structure generates qualitatively stronger opportunities for social bonding, shared identity formation and mutual regulation. Its particular cultural salience in Spain means that participants bring pre-existing emotional investments and social identities to the activity, amplifying its psychosocial effects relative to sports with lower cultural embeddedness. Furthermore, because football is a widely shared social practice outside prison, the social identities and competencies developed within the programme carry transfer potential to participants’ communities post-release, a feature that individually oriented exercise modalities cannot replicate.

The second emergent theme, *Personal Development*, encompasses two interconnected processes: growth and integration. The growth sub-theme reflects experiences of progressive mastery, overcoming challenge and sustained commitment that are consistent with SDT account of competence need satisfaction (Ryan and Deci, 2000). Participants described trajectories of skill development and growing confidence (from initial embarrassment to self-assurance) that align with Parker *et al.*’s (2014) qualitative findings in a young offender institution, where sport participation similarly facilitated the development of self-concept and social skills through progressive challenge. When interpreted through the Sport-Based Prisoner Rehabilitation Model (Morgan *et al.*, 2020), these experiences represent the accumulation of psychological capital (specifically, self-efficacy and resilience) and human capital (cooperation and communication) that the model identifies as central mechanisms of sport-based rehabilitation. Research by Liguori and Calella (2024) and De Marco and Meek (2022) similarly documents how structured sport in custodial settings promotes self-esteem and motivation for personal change, reinforcing the present findings.

The integration sub-theme warrants particular attention. Participants’ accounts of being treated as footballers rather than as institutional numbers reflect a symbolic shift in social role that carries substantial psychological significance. Social Identity Theory (Tajfel and Turner, 1979) helps explain this process: membership in a valued social group (the football team) generates a collective identity that temporarily displaces the stigmatised institutional identity of the person living in prison. This identity process may represent an important feature of football-based programmes, given their inherently collective nature.

The accounts provided by female participants regarding the integration sub-theme deserve specific interpretive attention, even given the small size of the female subsample. Meek and Lewis (2014), in a qualitative study with 44 women across three English prisons, documented that sport served as a critical mechanism for self-esteem, social interaction and coping among women in custody, a population for whom access to organised physical activity is structurally more constrained than for their male counterparts. The present study’s female participants echoed these findings, describing football as one of very few opportunities for organised sport and meaningful social interaction available to them within the facility. These observations are consistent with Peitz *et al.*’s (2025) findings from the Twinning Project, where women similarly emphasised the rarity of opportunity and the symbolic significance of being included. Taken together, these findings suggest that for women in custodial settings, the integration function of football carries a qualitatively distinct weight, one that future research with adequately powered female samples should examine in depth.

The third and final theme, *Environment*, is analytically the most distinctive contribution of the study. It encompasses two interconnected sub-themes, conditions and rituals, that together reveal how the football programme shaped participants’ experience of institutional time and space in ways that extended well beyond the sessions themselves.

The conditions sub-theme reflects participants’ experience of the football pitch as a qualitatively distinct space within the prison, one associated with agency, social openness and temporary freedom from the module’s constraints. Norman and Andrews (2019) conceptualised this dynamic as the “folding” of sport space into carceral space: sport participation does not simply add a recreational layer to the institution but produces an alternative spatial reality within it, enabling people living in prison to assert a limited degree of agency over their daily lives. In the present study, the transition from module to football pitch (even as a weekly 90-min event) constituted a meaningful spatial interruption to the otherwise continuous experience of confinement. Participants’ accounts of the scarcity of alternative activities reinforce this interpretation: the programme’s value was amplified by the institutional context in which it operated, consistent with Gallant *et al.*’s (2015) finding that sport provides a positive aspect in an otherwise negative environment.

The rituals sub-theme warrants particular interpretive attention because it highlights an aspect of football participation that has received relatively little attention in previous qualitative research. The pre-session behaviours participants described, preparing clothing the night before, experiencing heightened anticipation since Tuesday for a Thursday session, are not merely habitual acts. Drawing on Norman’s (2020) theoretical analysis of time in carceral spaces, these practices constitute temporal markers that restructure how institutional time is lived. People living in prison experience time simultaneously as chronological duration and as experiential flow shaped by the monotony of daily routine (Moran, 2012; Norman, 2020). Structured activities that create recurring positive anticipation interrupt this monotony and generate what participants described as a qualitatively different weekly rhythm, one organised around an anticipated event rather than the undifferentiated passage of institutional days. These accounts suggest that anticipation itself formed an important part of participants’ experience, extending the programme’s psychosocial significance beyond the sessions themselves. This finding may have important implications for programme design: interventions that create predictable, meaningful temporal landmarks within the prison week may generate psychosocial benefits that far exceed what session frequency alone would predict.

A further dimension of the Environment theme concerns the conduct-contingent nature of programme access. Participants who did not maintain appropriate institutional behaviour were excluded from sessions, a structural feature documented across multiple prison sport programmes internationally (Gallant *et al.*, 2015; Norman, 2020; Parker *et al.*, 2014). While this conditionality may incentivise prosocial behaviour, it also creates a tension: individuals may be excluded from the programme at precisely the moments when therapeutic engagement would be most beneficial. This tension is directly relevant to the Spanish penitentiary context, where the LOGP (1979) establishes rehabilitation rather than punishment as the primary objective of custodial sentences. Programme administrators should consider whether conduct-contingent access is consistent with this rehabilitative mandate, and whether alternative mechanisms for promoting prosocial behaviour might preserve therapeutic access for those who most need it.

The findings carry several implications for practice across three levels: programme design, institutional policy and research priorities.

At the level of programme design, the present findings suggest that the psychosocial benefits of football in custodial settings are closely tied to the conditions of participation rather than to technical content. Three specific recommendations follow. First, session frequency should be sufficient to sustain motivational engagement: participants explicitly identified one weekly session as insufficient, and the intensity of psychosocial need documented here suggests that more frequent contact may further enhance participants’ experiences. Second, facilitators should possess psychosocial competencies alongside technical coaching skills. The relational quality of the coach–participant interaction emerged as central to participants’ experiences of competence and recognition, consistent with Murray *et al.*’s (2024) international consensus recommendation that sport-based intervention facilitators receive training in psychosocial as well as technical domains. Third, gender-specific provisions should be incorporated into programme design. The present findings, consistent with Meek and Lewis (2014) and Peitz *et al.* (2025), indicate that women’s experiences in mixed football programmes have a qualitatively distinct character centred on access, recognition and dignity that mixed-provision models alone may not adequately address.

At the level of institutional policy, the conduct-contingent access structure documented in this study warrants critical examination. As noted above, excluding participants from a therapeutic programme on behavioural grounds risks removing access at precisely the moments when engagement may be most needed. Correctional administrations in Spain should consider whether this practice is consistent with the rehabilitative mandate established in the LOGP (1979), and whether alternative mechanisms for promoting prosocial behaviour could preserve therapeutic access for those most in need.

At the level of research priorities, three directions emerge from the present findings. First, a dedicated qualitative study of women’s football experiences in Spanish custodial settings, with a sample adequate for gender-specific analysis, is needed. Second, longitudinal designs are required to examine whether the psychosocial gains documented here persist post-release and contribute to desistance outcomes. Third, comparative studies across facilities with different security levels and institutional cultures would test the extent to which the present findings generalise beyond the medium-security context studied here.

This study has several limitations that should be considered when interpreting the findings. First, the study was conducted in a single medium-security correctional facility in Spain, which limits the transferability of findings to other correctional contexts, including closed-regime facilities, high-security establishments, or centres operating under different institutional cultures or national policy frameworks.

Second, the qualitative design, while generating contextually rich data, involved a relatively small sample drawn from a single programme. Although analytical saturation was reached, the findings reflect the experiences of a specific group of participants within a specific institutional setting and should not be generalised beyond comparable contexts.

Third, the perspectives of key stakeholders beyond participants were not captured. The views of programme facilitators, correctional officers and administrative staff were not included. Incorporating these perspectives in future research would provide a more comprehensive understanding of how sport-based programmes operate within the broader institutional environment.

Fourth, the dual role of one member of the research team, who served both as programme deliverer and as a co-analyst, represents a positionality consideration that, despite the mitigation strategies described in the Data Analysis section, may have influenced the interpretive process in ways that cannot be entirely eliminated.

Fifth, the small number of female participants (*n* = 3) means that the study was not designed to support gender-comparative analysis. The accounts provided by female participants offer valuable contextual insight into how the programme was experienced by women within this facility, but should be interpreted as preliminary and hypothesis-generating rather than as evidence of gender-specific patterns. Future research should examine women’s experiences of football in Spanish custodial settings using samples of sufficient size to support gender-specific analysis.

Finally, the study captured participants’ experiences at a single point during the programme. Longitudinal designs would enable examination of how psychosocial experiences evolve over time and whether gains observed during participation persist following release.

## Conclusions

This study examined the subjective experiences of people living in a Spanish medium-security correctional facility who participated in a structured football programme across a full academic year. The analysis identified three interconnected themes (wellbeing, personal development and environment) through which football participation operated as a meaningful psychosocial resource within the custodial setting.

The findings suggest that football’s contribution in this context operated not primarily through its technical content but through its structural role as a distinctive temporal, social and symbolic space. Participants described experiences of psychological regulation, progressive mastery, social recognition and ritualistic anticipation that extended the programme’s psychosocial reach well beyond the sessions themselves. These processes are consistent with Self-Determination Theory, Social Identity Theory and the Sport-Based Prisoner Rehabilitation Model, and extend the existing qualitative literature on sport-based programmes in correctional settings to the Spanish penitentiary context, a setting that has received virtually no attention in the international literature.

The study makes three main contributions. First, it provides qualitative evidence of how a football programme operates as a psychosocial resource in a Spanish custodial context, situating these findings within the constitutional and statutory rehabilitative mandate of the Spanish penitentiary system. Second, it demonstrates the analytical value of combining individual interviews, focus groups and non-participant observation in prison sport research, with each method contributing distinct and complementary evidence. Third, it offers preliminary evidence of gendered differences in how football participation is experienced within a mixed custodial programme, underscoring the need for dedicated research on women’s sport experiences in Spanish correctional settings.

Practically, the findings support the inclusion of structured football programmes within prison rehabilitation frameworks, particularly when facilitators are trained in psychosocial as well as technical competencies, session frequency is sufficient to sustain meaningful engagement, and programme access is decoupled from purely punitive conduct management mechanisms. Such initiatives, grounded in the rehabilitative philosophy of the Spanish penitentiary system, have the potential to contribute to wellbeing, social integration and the conditions that support desistance from crime.
